# Effect of Fibre Orientation on the Quasi-Static Axial Crushing Behaviour of Glass Fibre Reinforced Polyvinyl Chloride Composite Tubes

**DOI:** 10.3390/ma14092235

**Published:** 2021-04-27

**Authors:** Rahib A. Khan, Elsadig Mahdi, John-John Cabibihan

**Affiliations:** Mechanical and Industrial Engineering Department, College of Engineering, Qatar University, Doha P.O. Box 2713, Qatar; rk1107460@student.qu.edu.qa (R.A.K.); john.cabibihan@qu.edu.qa (J.-J.C.)

**Keywords:** filament winding, fibre orientation, PVC, crashworthiness, crushing behaviour

## Abstract

In this study, glass fibre reinforced (GFRP) polyvinyl chloride (PVC) tubes were subjected to quasi-static axial compression tests to determine their crashworthiness performance. To this end, this study employed GFRP/PVC tubes with four different fibre orientations, 45°, 55°, 65° and 90°. A five-axis filament winding machine was used to fabricate the tubes. The results show that there was a considerable increase in all crashworthiness characteristics due to GFRP reinforcement. For the GFRP/PVC composite tubes of different fibre orientations, the load-bearing capacity, crush force efficiency and energy absorption capability generally improve with increasing fibre orientation. The GFRP/PVC 45° specimen was a notable exception as it exhibited the best specific energy absorption capacity and a crushing force efficiency that was only slightly less than for the GFRP/PVC 90° specimen.

## 1. Introduction

In passenger vehicles, “crashworthiness” refers to a structure’s capacity to absorb the impact energy from collisions and, in so doing, increase the survivability of the occupants [1]. Crashworthiness is explicitly concerned with this absorption taking place through controlled failure mechanisms and modes to enable a stable load profile during the absorption process [2]. Crashworthiness is an integral part of vehicle safety in the automotive industry. Due to increasing concerns over the cost of fuel and automobiles’ environmental footprint, the automotive industry is also continuously seeking means to improve fuel efficiency, such as employing more advanced materials [3]. Approximately 75% of the fuel consumed by a vehicle is due to its weight. Estimates have been made that, by reducing a vehicle’s weight by about 10%, it would be possible to decrease its fuel usage by as much as 8%. This reduction translates into a significant drop in the amount of carbon dioxide generated by a vehicle over its lifetime [4]. Designers manufacture composites according to the application requirements.

Additionally, they have high stiffness to weight and strength to weight ratios and excellent fatigue and corrosion resistance. All of these properties make them very appealing as materials for crashworthiness applications [5]. A substantial proportion of composite applications involve glass fibre reinforced polymers (GFRPs). One of the main reasons for this is that composites with other reinforcing material types, such as carbon and aramid fibres, are often considerably more costly [6]. With suitable composition and fibre orientation, GFRP composites have a higher stiffness than aluminium and a specific gravity one-quarter of steel [7]. GFRPs have been used in automotive applications [8], aerospace applications [9], civil infrastructures [10], marine and piping industries [11]. The challenge in employing composites for crashworthiness is to use specific geometry and materials to facilitate better safety standards while minimising weight. Moreover, this should be done without exceeding what would be considered acceptable production costs [12].

The crashworthiness characteristics of collapsible composite energy absorber devices are significantly affected by several different factors. These factors include the selected constituent materials forming the composite and their properties, manufacturing conditions, geometry of the composite, loading conditions and environmental conditions [13]. Various studies have been conducted to investigate the crashworthiness properties of composite energy absorbers. Lui et al. [14] carried out quasi-static and dynamic axial compression tests on several circular carbon/epoxy composite tubes with varying thicknesses, layups, and composite prepreg sheet architectures. The results show that thicker specimens exhibited a higher specific energy absorption. Mirzaei et al. [15] experimentally and analytically investigated aluminium/epoxy hybrid circular tubes’ behaviour when subjected to quasi-static axial compressive loads. They found that the stacking sequence had a considerable effect. For some specimens, the hybrid tubes absorbed more than three times that of the aluminium tubes independently. Wang et al. [16] developed a reliability-based design optimisation approach to examine the effect of variable angle tow composite cylinders. Filament winding was selected as the preferred manufacturing method as this modern fabrication process allows for the fibre orientation to be easily varied during production. Additionally, filament winding has a high production rate, ensures excellent quality and can produce composite structures with a high fibre volume fraction. The results show that variable angle tow composite cylinders could outperform their constant stiffness counterparts. Almeida Jr. et al. [17] investigated filament wound carbon fibre/epoxy composite tubes’ failure under axial compression. Additional hoop layers were added to the ends of the specimens to reinforce them. Analytical and linear numerical models were also used to predict the failure behaviour of the tested samples. The results show that the thinner tested tubes underwent buckling failure while the thicker tubes exhibited transverse shear failure. Alkateb et al. [18] experimentally examined the effect of changing the vertex angle on the energy absorption capability of axially crushed kenaf/epoxy composite elliptical. The vertex angle of the manufactured tubes was varied from 0° to 24° in 6° increments, where the elliptical cone with a vertex angle of 0° was an elliptical tube. They found that that load carrying capacity and energy absorption capability generally increased with increasing vertex angle. Palanivelu et al. [19] carried out quasi-static compression experiments on E-glass fabric/polyester composite tubes with nine different geometries, including varied thicknesses and different triggering mechanisms. They found that the conical and circular composite tubes exhibited the best load carrying capacity and energy absorption capability. Chiu et al. [20] focused on the experimental investigation of a carbon/epoxy composite tube’s energy absorption behaviour under quasi-static and dynamic loading with varying strain rates not exceeding 100 s^−1^. Analysis of the results indicated that the tested composite tubes were strain rate-independent for strain rates not exceeding 100 s^−1^. They concluded that this independence rate could be attributed to the composite using a high fibre volume fraction. Eggers et al. [21] examined the effect of fibre orientation angle, stacking sequence and diameter to thickness ratio on the mechanical properties of filament wound carbon fibre/epoxy composite rings subjected to three different loading conditions, respectively. The tested loading conditions were radial compression, axial compression and hoop tensile loading. For axial compression, it was found that the axial compressive strength of the composite rings increased with decreasing fibre orientation. Conversely, the axial compressive strength of the composite rings increased with increasing diameter to thickness ratio.

Considerable research has focused on the effect of fibre orientation on the crashworthiness characteristics of fibre-reinforced composites. Ozbek et al. [22] investigated the crashworthiness characteristics of intra-ply hybridised composite tubes manufactured by combining basalt and glass fibre reinforcements with three different fibre orientation angles. Their results show that the basalt/epoxy composite tubes had the highest crush force efficiency, while the glass/epoxy samples had the best specific energy absorption values. Furthermore, they found that, with an increase in fibre orientation angle, there was a corresponding drop in energy absorption and a rise in crush force efficiency. Hu et al. [23] experimentally examined the effect of fibre orientation angle on composite circular tubes’ crashworthiness properties manufactured from 759/5224 woven glass cloth/epoxy. They chamfered all the tested tubes with an external angle of 45°. For both the quasi-static compression and impact tests, the peak load initially reduced with increasing fibre orientation from 15° to 45° but subsequently increased with rising fibre orientation from 45° to 75°. The composite tube with a fibre orientation of 45° had the smallest SEA for both the quasi-static compression and impact tests, while the composite tubes with fibre orientation of 30° and 60° had comparatively high SEA. Jia et al. [24] systematically evaluated the effects of a geometric factor, fibre orientation angle and pre-crack angle on the energy absorption behaviour of filament wound CFRP circular composite tubes subjected to quasi-static axial crushing. The CFRP composite tubes with end reinforcing layers demonstrated high compressive characteristics. Mahdi et al. [25] carried out an experimental investigation to optimise the energy absorbed by axially crushed composite tubes. The circular composite tubes tested were manufactured using layers of E-glass woven fabric and epoxy resin. From the analysis of the overall results, the fibre orientations 15°/−75° and 75°/−15° had the best load capacity and energy absorption performance. Although researchers have carried out several studies on the effect of fibre orientation on axially crushed composite tubes’ crashworthiness characteristics, they are still insufficient. This finding is factual when considering the different constituent materials of composites and the varying manufacturing methods utilised in these investigations. Therefore, there is a need for more research to enrich scientific knowledge in this area. This paper aims to study fibre orientation’s influence on the crashworthiness properties of GFRP polyvinyl chloride (PVC) tubes subjected to quasi-static axial compression. Whereas some research has investigated the crashworthiness of composite tubes manufactured from GFRP on their own, studies on this particular material combination are comparatively scarce.

## 2. Materials and Methods

### 2.1. Materials and Geometry

**Figure 1:**
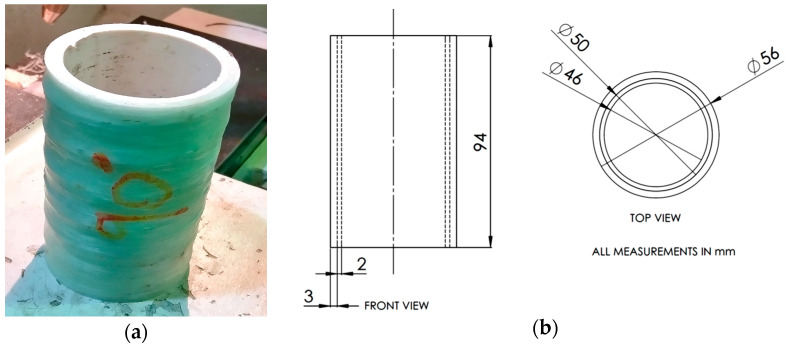

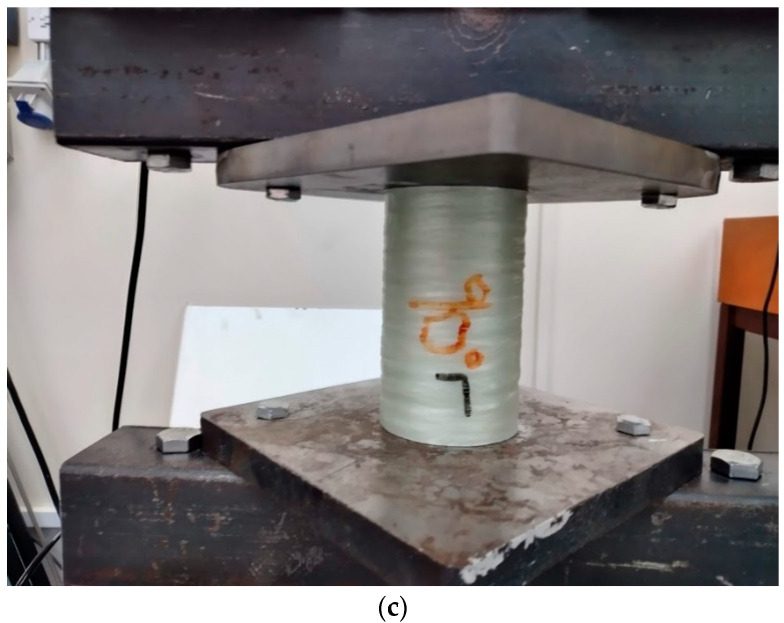
(**a**) Fabricated specimen; (**b**) schematic diagram of top and side views with average sample dimensions; (**c**) Sample positioned between compressive plates before the test.

GFRP was used to reinforce PVC tubes at four different fibre orienta-tions, 45°, 55°, 65° and 90°, where the reference axis 0° corresponds to the longitudinal ax-is of the tubes. The selection of the fibre orientation is random. The fabricated composite tubes were subjected to quasi-static axial compression tests to determine the effect of GFRP reinforcement and fibre orientation on their crashworthiness properties. The specimens prepared for compressive testing had a length of 94 mm. The PVC inner tube had an outer diameter of 50 mm and a thickness of 2 mm. The weight of the PVC inner tube on its own was about 0.038 kg. The thickness of the added glass fibre/epoxy reinforcement was an average of approximately 3 mm. Figure 1a,b show the GFRP/PVC sample being faced using a turning machine and the Schematic diagram of top and side views, respectively.

There were slight differences in the specimens’ thickness. Accordingly, the thickness of the 45° specimens was closer to 2.5 mm, with the thickness increasing for increasing fi-bre orientation angle up to approximately 3.5 mm for the 90° specimens. The average weights of the tested 45°, 55°, 65° and 90° specimens were approximately 0.098, 0.129, 0.142 and 0.143 kg, respectively. As a standard, three tests were carried out for each of the four winding angles. Figure 1c shows GFRP/PVC tube positioned between compressive plates before the test.

### 2.2. Manufacturing Process

The winding process was used to fabricate the specimens is an automated open moulding process that uses a rotating mandrel as the mould. The winding process results in a high degree of fibre loading, which provides high tensile strength in the manufacture of hollow and circular tubes. Continuous reinforcement was fed through a resin bath and wound onto a long PVC tube, placed over the rotating mandrel. For the fabrication of composite tube specimens for this study, glass fibre (Owens Corning, Toledo, OH, USA), in the form of E glass fibre rovings, was used as the reinforcing material, while epoxy resin was used as the matrix material. A helical filament winding pattern was used to manufacture the 45°, 55° and 65° specimens. On the other hand, a hoop filament winding pattern was used for producing the 90° specimens. As provided by the suppliers, the material properties of the constituent materials used to manufacture the tested composite tubes are shown in Table 1.

**Figure 2:**
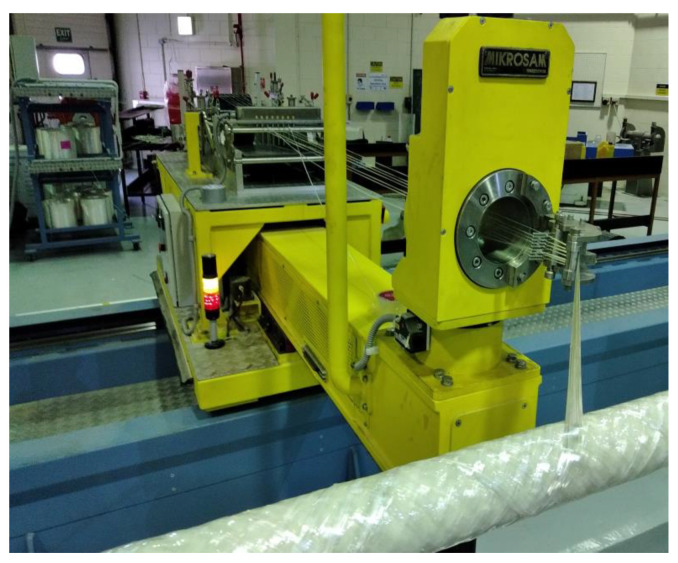
Fabrication of GFRP composite overwrapped plastic tube using five-axis filament winding machine.

Figure 2 shows the fabrication process. The ratio of hardener to epoxy resin was 30 parts to 100 parts. The reinforcement was laid down in a predetermined geometric pattern to provide maximum strength in the directions required. This process was used to rein-force long PVC tubes with glass fibre/epoxy at winding angles of 45°, 55°, 65° and 90°. For each angle, eight layers were applied before curing. Table 2 summarises the winding pa-rameters used to fabricate the GFRP/PVC tubes.

### 2.3. Test Setup

A quasi-static crushing test was carried out using the INSTRON material testing machine (Instron, Norwood, MA, USA). Before each test started, the upper and lower plates were positioned to contact the tested specimen’s upper and lower surfaces, respectively. During the crushing test, the lower plate of the testing machine was kept static/stationary while the upper plate moved downwards at a constant speed. All specimens were crushed at a 500 mm min^-1^ speed up to a displacement of 75 mm. The PVC specimens were an exception since they experienced material densification at a displacement of over 75 mm. The maximum capacity of the machine used was 250 kN. The machine’s computerised data acquisition system automatically recorded the loading and displacement values. Videos of the crushing process for all specimens under compressive loading were recorded to provide the crushing process’s history and allow the crushing mechanisms to be observed. Finally, small square samples were cut from the topmost side section of the crushed composite tubes. Scanning electron microscope (SEM) images were captured from these samples. The test setup is shown in Figure 1c

### 2.4. Crashworthiness Performance Indicators

**Figure 3:**
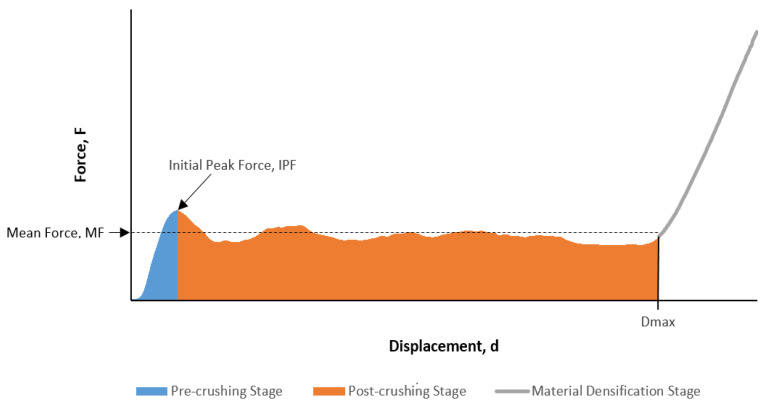
Typical load-displacement curve for the composite tube.

The assessment of the crashworthiness performance of composite structures can be carried out qualitatively and quantitatively. Qualitative assessment includes the structure’s overall crushing behaviour, the composites’ force–displacement properties and failure mechanisms exhibited by the structure. Quantitative analysis includes quantifying crashworthiness characteristics that allow one to have an idea of the structure’s energy-absorbing capabilities [26]. Some essential parameters that reflect a structure’s crashworthy characteristics are detailed below. Figure 3 shows some of the necessary quantities that are needed from the load–displacement curve while calculating these parameters.

#### 2.4.1. Initial Peak Force

The initial peak force (IPF) is the force needed to commence plastic tube deformation. The initial peak force should not be high as this will help reduce the possibility of passenger injury by lowering the size of reaction forces experienced in situations where safety is essential such as car and train collisions [27].

#### 2.4.2. Energy Absorption and Specific Energy Absorption

Energy absorption is quantified to evaluate composite structures’ ability to absorb crushing energy that results from collisions. This quantity is represented graphically as the sum of the areas under the force-displacement curve for the pre-crush and post-crush regions, i.e., the shaded area in Figure 3. In mathematical terms, the energy absorbed (EA) is represented as follows Equation (1) [28]:

(1) $$\mathrm{EA}=\int_{0}^{\mathrm{Dmax}}\mathrm{Fds}.\;$$

Here, F (N) is the compressive load. The specific energy absorption (SEA) gives a measure of the absorbed energy about the mass of the structure, Equation (2):

(2) SEA =EAm. 

where m (kg) is the mass of the structure under compression.

#### 2.4.3. Mean Crushing Force

The mean crushing force (MCF) is the average force that a composite structure experiences under compression. This quantity is calculated while excluding forces experienced in the densification stage Equation (3) [29]:

(3) MCF =EADmax. 

Here, Dmax (mm) is the displacement of the composite structure at the beginning of the densification stage.

#### 2.4.4. Crush Force Efficiency

Crush force efficiency (CFE) quantifies the ratio of the mean force experienced by the composite and to its initial peak crushing force Equation (4) [26]:

(4) CFE =MCFIPF. 

An ideal situation in terms of energy absorption is achieved when CFE has a value of 1, which means that the initial peak crushing force would be sustained through the entire post-crushing stage so that it is approximately equal to the mean crushing force.

## 3. Results and Discussion

**Figure 4:**
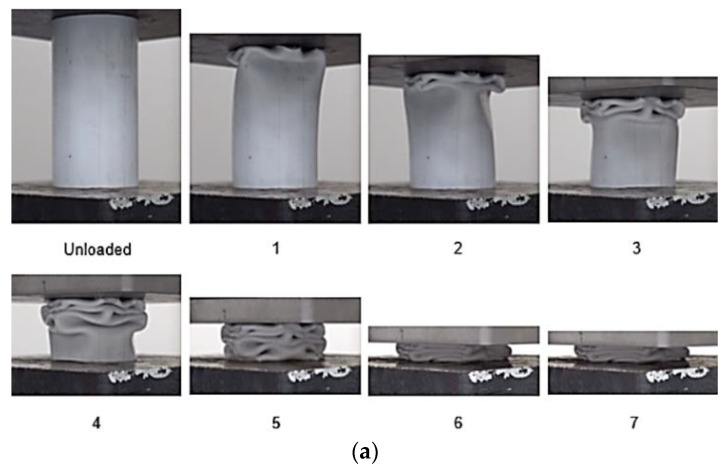

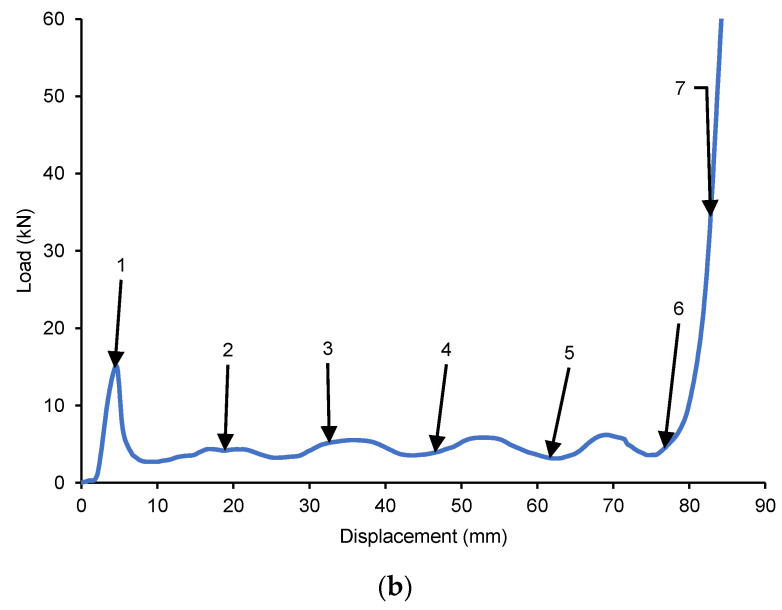
(**a**) Crushing history for PVC (**b**) Load–displacement curve for axial compression test of PVC sample.

**Figure 5:**
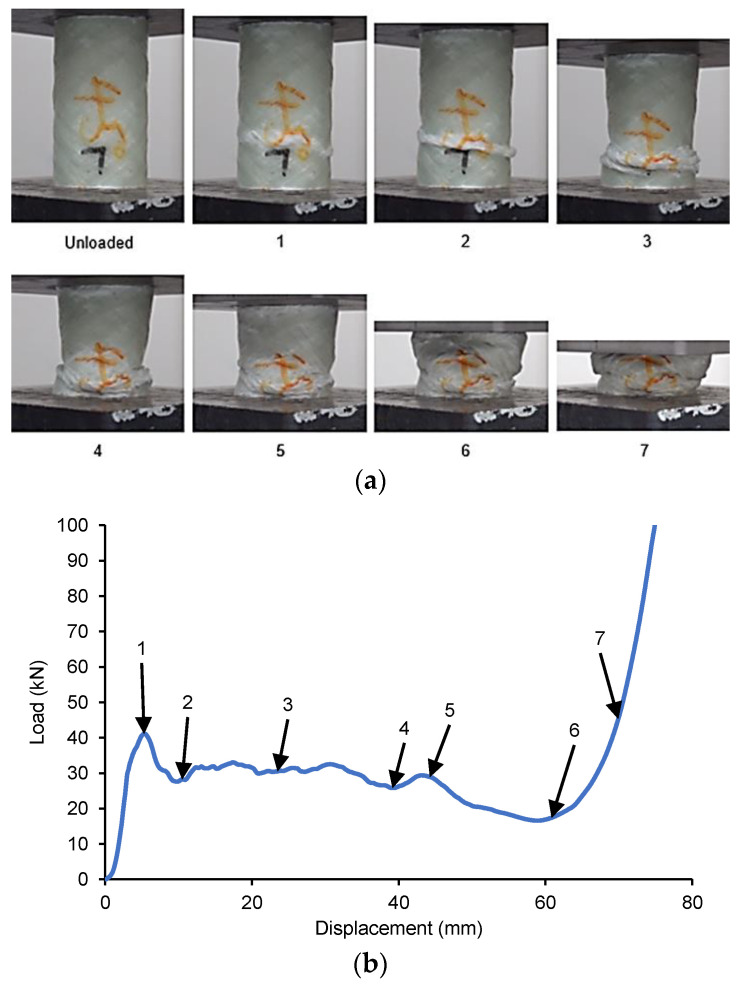
(**a**) Crushing history for GFRP/PVC 45° (**b**) Load–displacement curve for axial compression test of GFRP/PVC 45° sample.

**Figure 6:**
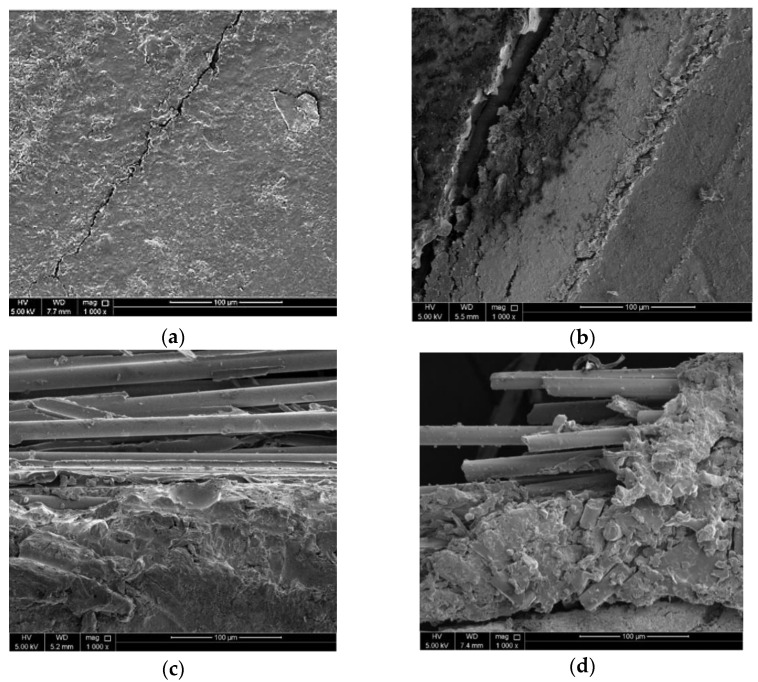
SEM images of side surfaces of GFRP/PVC composite tubes after crushing with fibre orientation angles of: (**a**) 45°; (**b**) 55°; (**c**) 65°; and (**d**) 90° (1000× magnification).

**Figure 7:**
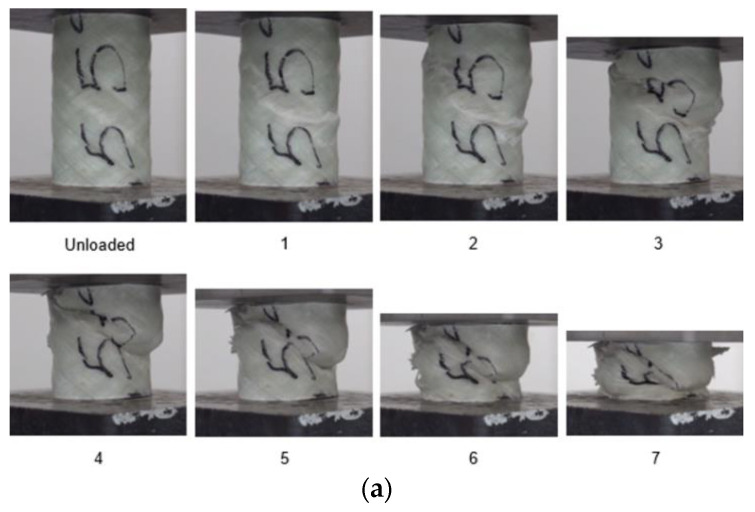

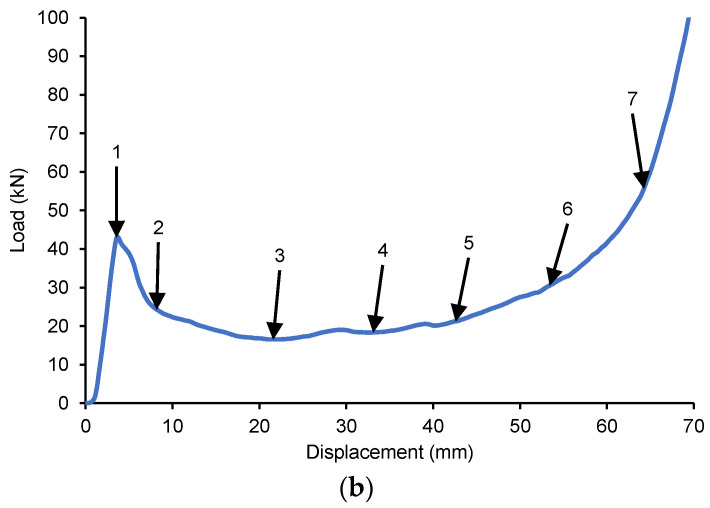
(**a**) Crushing history for GFRP/PVC 55° (**b**) Load–displacement curve for axial compression test of GFRP/PVC 55° sample.

**Figure 8:**
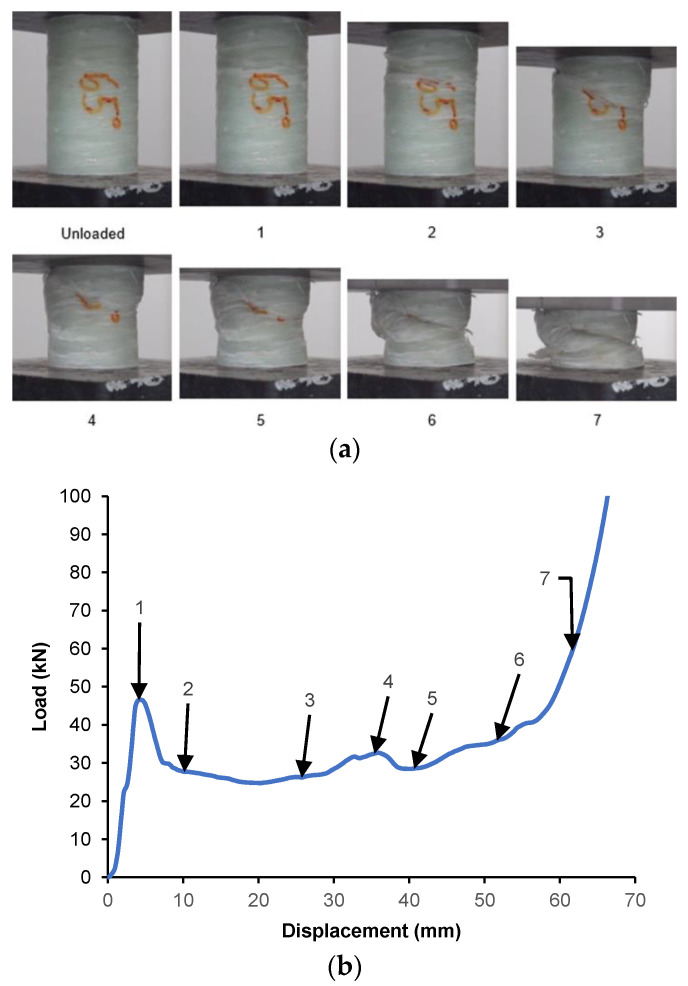
(**a**) Crushing history for GFRP/PVC 65° (**b**) Load–displacement curve for axial compression test of GFRP/PVC 65° sample.

**Figure 9:**
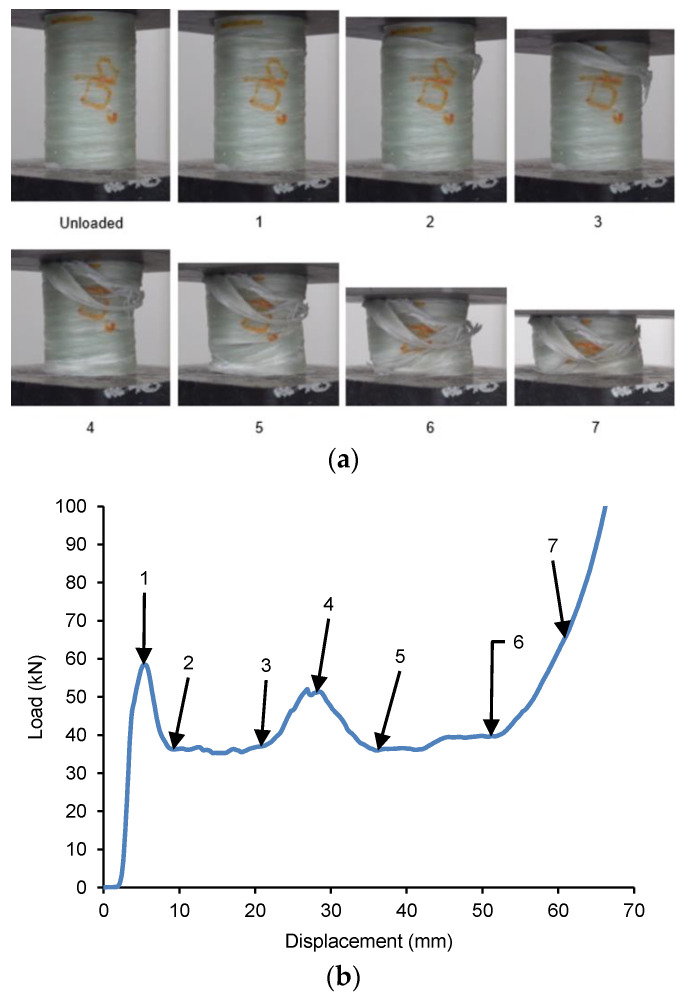
(**a**) Crushing history for GFRP/PVC 90° (**b**) Load–displacement curve for axial compression test of GFRP/PVC 90° sample.

**Figure 10:**
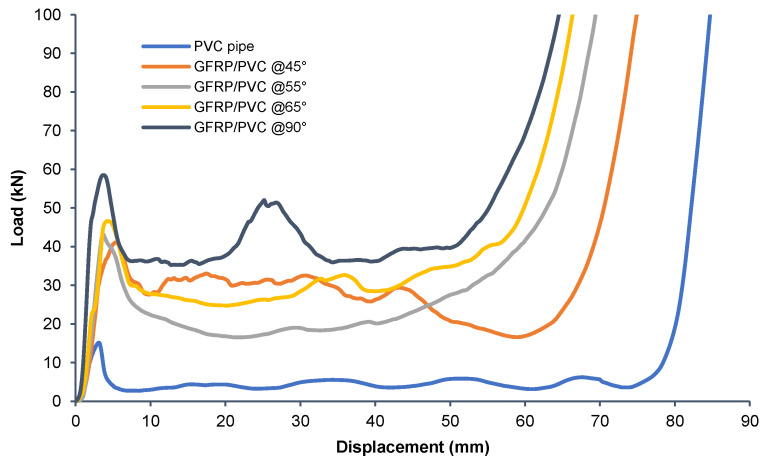
Load–displacement curves for axial compression tests of all GFRP samples vs. PVC tube.

**Figure 11:**
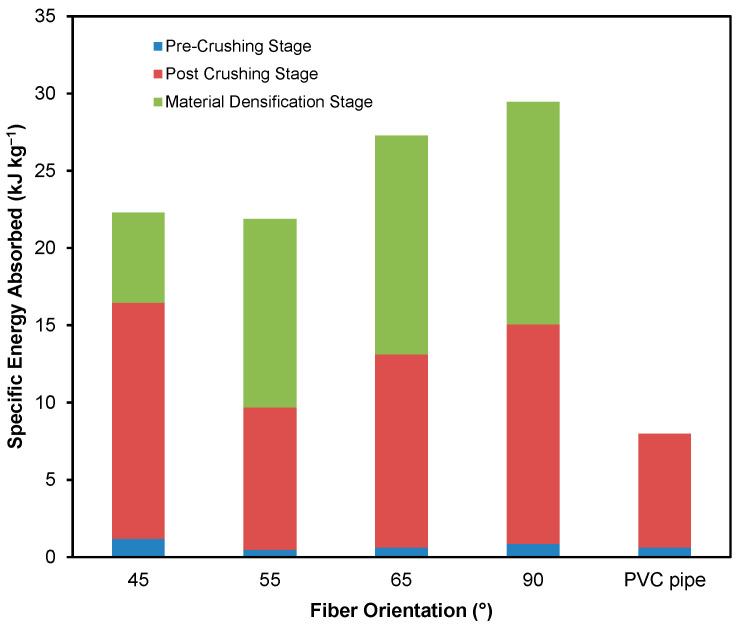
Specific energy absorbed by the GFRP PVC tubes and the PVC tube on its own.

In this section, the results of the quasi-static axial compression tests carried out on the various composite tubes are categorised, presented and discussed. Figure 4, Figure 5, Figure 6, Figure 7, Figure 8, Figure 9, Figure 10 and Figure 11 show the load–displacement curves, energy absorption graph and SEM images of fractured surfaces. The effect of reinforcing the PVC tubes with GFRP and changing the fibre orientation on the load-bearing behaviour, crush force efficiency and the composite tube specimens’ energy absorption capability is examined in detail.

Generally, the load–displacement curves exhibit some similarities for all composite tubes, as illustrated in Figure 3. Initially, the load–displacement curve will have a straight-line region for which the load increases in direct proportion to the displacement of the composite tube, referred to as the elastic region. For composite testing, the pre-crash stage includes the elastic region and ends at the displacement corresponding with the first turning point of the graph, at which the specimen experiences its first drop in load-bearing capability. The maximum load reached before this drop is the initial peak force. The region starting from the displacement where the initial peak force occurs up to the displacement at which densification begins is commonly referred to as the post-crash stage. After the post-crash stage, the load values increase dramatically in the densification stage. For all quasi-static axial compression tests conducted, videos were recorded and some images corresponding to the load–displacement behaviour of the tested composite tubes were extracted to assist in identifying the failure modes that the specimens experienced.

### 3.1. Failure Modes

According to Mamalis et al. [30], the failure modes commonly explicitly observed in thin-walled cylindrical composite tubes when subjected to quasi-static axial compression can be broadly divided into three modes, referred to as Modes I–III. In Mode I, failure occurs due to the progressive crushing, accompanied by micro fragmentation, of the composite tube. The distinguishing trait of this particular failure mode is long interlaminar, intra-laminar and axial cracks that exceed the length of the composites’ laminate thickness. These cracks divide the fibres into bunches, commonly known as fronds [31]. These fronds spread radially outwards and inwards from the surface of contact between the composite tube and the compression platen, causing the appearance of the crushed cylindrical composite sample to resemble a mushroom. The curved fronds’ radius changes depending on the composite’s fibre, matrix and laminate properties. Sigalas et al. [32] identified the forces that emerge in the crush zone in the course of frond formation as follows: compressive loads experienced by fronds and debris wedge, abrasion forces as the fronds scrape across the load plates, abrasion forces between the fronds and the debris wedge, abrasion forces that exist between the lamina of the composite and fronds as they twist through the radii of curvature and hoop forces that occur as a result of the inner and outer fibres attempting to resist longitudinal crack propagation. There are several means by which composites that experience this failure mode attempt to dissipate the resulting energy, including energy absorbed due to the formation of cracks in the longitudinal wall, energy absorbed due to delamination and the process of forming fronds, energy used in curving the fronds, energy absorbed due to the fracture of fibres, energy used up by the numerous forms of abrasion mentioned above and, finally, energy needed to form additional cracks, such as those resulting from axial tube splitting [33]. In existing studies, the amount of energy dissipated due to each process is not clear, although it is known that the numerous abrasion forces account for as much as half of the total energy dissipated due to Mode I failure [34]. In this study, three failure modes were identified as follows:

#### 3.1.1. Mode I

In this mode, failure occurs due to the progressive crushing, accompanied by micro fragmentation, of the composite tube. This mode of failure is usually characterised by the formation of continuous fronds, which spread radially outwards and inwards in the form of a mushrooming failure. Of the three failure modes described, Mode I was identified as the failure mode that results in the highest amount of energy being absorbed during crushing.

#### 3.1.2. Mode II

This mode associated with failure occurs due to brittle fracture of the composite tube. This failure mode may be initiated by either longitudinal or circumferential crack propagation. Transverse shear is also usually a contributing factor. In some cases of failure due to transverse shear, complete separation across the fracture plane does not occur. Further loading may result in the interpenetration of the two halves of the composite tube on either side of the fracture plane. This interpenetration, in turn, can give the composite tube some residual load-bearing capacity. Mode II is the failure mode associated with the least energy absorption of the three failure modes specified.

#### 3.1.3. Mode III

Finally, in Mode III, failure occurs due to progressive folding and hinging, similar to thin-walled metal and plastic tubes’ crushing behaviour. When compared to Modes I and II, Mode III exhibits a medium energy absorbing capacity. Figure 4, Figure 5, Figure 6, Figure 7 and Figure 8 show that, comparatively, for the displacement for which the GFRP/PVC 45° composite tube exhibited Mode III failure, its load-bearing capability was higher than that of the GFRP/PVC 55° and GFRP/PVC 65° samples, which exhibited Mode II failure. The composite tube that undergoes Mode III failure will have a higher load-bearing capacity and energy absorption ability than one which undergoes Mode II failure. The GFRP/PVC 90° sample was an exception in this case as, although it exhibited Mode II failure, it had the highest load-bearing capacity of the tested samples. This finding can be attributed to the fibre orientation of the GFRP/PVC 90° composite tube. As the loading direction was normal to the fibre orientation, the fibres provided a higher resistance to loading. This load-bearing behaviour suggests that the tested composite tubes had a reasonably high fibre volume fraction, as only with a high fibre volume fraction would the spacing between the fibres in the GFRP/PVC 90° sample be small enough to allow for the fibres to bundle together and support each other effectively when subjected to compressive loading. It is also observed that the GFRP/PVC 55°, GFRP/PVC 65° and GFRP/PVC 90° composite tube samples experienced transverse shear failure to different degrees. The load-bearing behaviour of these samples indicated that, with a decrease in the degree of transverse shear failure, there was a corresponding increase in load-bearing capacity. It is also seen that the matrix cracks formed the transverse fracture planes for each of these samples, respectively, mainly occurred along the direction of fibre orientation. On the other hand, crack propagation perpendicular to the fibre was inhibited effectively by the fibres. Another notable observation was that none of the tested specimens experienced Mode I failure, which can be due to the strong interface between the GFRP and the PVC tube. These two factors prevent the formation of fronds while seemingly promoting the occurrence of Mode III failure. On the other hand, the PVC inner tubes also reduce the severity of brittle fracture when Mode II failure occurs; additionally, compared to composite tubes that undergo Mode II failure, the crushed samples’ fragmentation was not as severe.

### 3.2. Load–Displacement and Visual Observation

#### 3.2.1. PVC Tube on its Own

To determine the effect of using GFRP to reinforce the PVC tubes used as the frame for the composite tubes, it was necessary to first observe the load behaviour of a PVC tube on its own, with the PVC tube having dimensions identical to those used in the composite tubes for the crushing tests. Figure 4 shows the load–displacement curve for the PVC tube and the corresponding images. Figure 4 shows that, initially, the graph has a straight-line region, where load increases in direct proportion to the displacement of the composite tube. The load eventually reaches a local maximum, the initial peak load, after which the load drops noticeably. For the PVC tube, the initial peak force had a value of approximately 15.14 kN and occurred at a displacement of about 4.599 mm. The images in Figure 4 show that the failure mechanism for the PVC tube was progressive folding. The progressive failure was expected as plastic tubes typically undergo progressive folding under compression, as is characteristic of Mode III failure. The compressive stress exerted on the PVC tube increased until it was large enough that it caused the tube wall to buckle locally and form a hinge. It was observed that the formation of the first hinge in the PVC tube happened simultaneously as the initial peak force was reached. After this, as the first fold formed, quite a considerable drop in load-bearing capability occurred as it dropped from 15.14 kN down to about 2.732 kN. Following this, folds continued to form, and, for each fold, stresses built up in the tube wall, forming a hinge, followed by a subsequent drop in the load-bearing capacity as folding occurred. However, for the second fold onwards, the load’s rise and fall were not as high, and the failure was more stable as a result. The region starting from the displacement where the initial peak force occurred, 4.599 mm, up to the displacement at which densification began for the PVC tube, 77.56 mm, is the post-crush stage for the specimen. For the post-crush stage of the PVC tube, the average load was approximately 4.397 kN. After the displacement value of 77.56 mm, the load value increased drastically.

#### 3.2.2. GFRP/PVC tube with 45° Fibre Orientation

Figure 5 shows the load–displacement graph for the GFRP/PVC 45° composite tube subjected to quasi-static axial crushing and the images corresponding to the labelled points in the graph. It can be seen that the failure mode first exhibited by this sample was Mode III failure as the initial failure of the tube occurred due to local buckling, with a hinge forming at the initial peak force of about 41.11 kN at a displacement of 5.409 mm. Once this fold formed fully, the load reached a local minimum of approximately 27.60 kN, while the displacement at this point was about 10.93 mm. After this, until a displacement of approximately 44.09 mm, the GFRP/PVC 45° composite tube continued to display Mode III failure as the load fluctuated about a mean value of 27.16 kN and a second fold formed in the composite tube. Subsequently, however, the top of the tube collapsed into itself, resulting in a noticeable drop in load-bearing capacity to a new overall minimum of 16.59 kN at a displacement of approximately 59.01 mm and severe matrix cracking. This drop in load-bearing capacity bore a closer resemblance to the kind of behaviour that would be expected of Mode II brittle failure. As shown in Figure 5, the GFRP/PVC 45° composite sample exhibited Mode III failure until a displacement of about 44.09 mm. After this, the failure mode changed to Mode II failure until, finally, the composite tube load rose steeply as the composite tube was compacted in the densification stage.

Although the damage to the GFRP/PVC 45° composite tube due to buckling, matrix cracking and the final collapse of the sample was considerable, it was seen that the adhesion between the GFRP and the PVC inner tube remained as the two components of the composite tube did not separate. Moreover, delamination during failure was negligible as even after densification; there were no noticeable hanging loose bunches of the GFRP. This observation was confirmed by examining a part of the sample, after crushing, that was not significantly affected by buckling and matrix cracking using an SEM. From the SEM image of a section of the GFRP/PVC 45° composite tube after crushing (see Figure 6a), it can be seen that the GFRP layers were relatively intact. Although matrix cracking occurred, the cracking severity was not as pronounced as for the other samples.

#### 3.2.3. GFRP/PVC Tube with 55° Fibre Orientation

Figure 7 shows the load–displacement graph for the GFRP/PVC 55° composite tube when subjected to quasi-static axial crushing and the images corresponding to the points labelled in the graph. During the pre-crush stage, the top section of the tube first shifted noticeably towards one side due to transverse shear failure. During this transverse shear failure, longitudinal cracking was almost negligible, and the cracks formed were predominantly along the angle of fibre orientation.

The transverse shear was not sufficient to cause complete separation across the fracture plane, and, eventually, the interpenetration of the two halves of the tube on either side of the fracture plane occurred at the initial peak force of 43.21 kN and a displacement of 3.666 mm. The two halves of the tube then continued to interpenetrate through one another, causing a considerable drop in load-bearing capacity and significant matrix cracking. This type of catastrophic failure is characteristic of Mode II failure. The bearing capacity reached a minimum of about 16.56 kN at a displacement of approximately 22.43 mm due to this. After this, the fold due to interpenetration enlarged, resulting in the matrix cracks lengthening noticeably. While the fold was enlarged, loading was relatively stable until a displacement of about 40.10 mm. Then, the interpenetration fold collapsed, and the two halves of the specimen began compacting together accompanied by a rise in load. Finally, the compaction of the composite tube continued throughout the densification stage. The compaction at the end of the composite tube failure caused noticeable delamination and fibre pull-out. From the SEM image of a section of the GFRP/PVC 55° composite tube after crushing (see Figure 6b), the extent of matrix cracking was noticeably more extensive than for the GFRP/PVC 45° sample, as evidenced by the more significant number of cracks and larger crack width.

#### 3.2.4. GFRP/PVC tube with 65° Fibre Orientation

Figure 8 shows the load–displacement graph for the GFRP/PVC 65° composite tube when subjected to quasi-static axial compression and the images corresponding to the points labelled in the graph. At first, due to the build-up of transverse shear stress, the top third of the composite tube specimen shifted slightly to the side, and a fracture plane was formed due to circumferential cracking. The fracture plane fully formed at the initial peak load of 46.62 kN, which occurs at a displacement of about 4.245 mm. The two sections of the tube then continued to interpenetrate through one another, while the matrix cracks caused by the split also lengthened, resulting in the tube’s load-bearing capacity decreasing. Initially, the load dropped quite steeply until it reached a value of about 29.90 kN at a displacement of 7.625 mm. This type of catastrophic drop in load-bearing capacity is characteristic of Mode II failure.

After this, the specimen’s load-bearing capacity decreased until the load reached a local minimum of approximately 24.77 kN, at a displacement of 19.76 mm. Then, another small split due to circumferential cracking formed in the bottom third of the composite tube. As the split formed, the specimen’s load-bearing capacity also increased until a fracture plane fully formed at a local maximum load of about 32.61 kN, which occurred at a displacement of 36.10 mm. Again, the two newly formed sections on either side of the fracture plane interpenetrated one another, causing a slight drop in load-bearing capacity, up to a local minimum of 28.45 kN, which corresponded to a displacement of 40.01 mm.

The fold formed, and the three sections of the tube began to compact together. This compaction gives rise to an increase in the load-carrying capacity. This compaction continued throughout the densification stage; the rate at which the load-bearing capacity was rising increased as compaction progressed; and a noticeable amount of delamination also occurred. From the SEM image of a section of the GFRP/PVC 65° composite tube after crushing (see Figure 6c), one can notice the matrix cracking and delamination that occurred. The bottom of the captured image shows the surface of the composite sample that is relatively smooth, while the top of the captured image shows the glass fibres that were exposed due to delamination.

#### 3.2.5. GFRP/PVC tube with 90° Fibre Orientation

The load–displacement behaviour for the GFRP/PVC 90° composite tube when subjected to quasi-static axial compression is shown in Figure 9, along with the images corresponding to the points labelled in the graph. As the compression test for this composite tube progressed, due to the build-up of buckling stresses in the composite tube, circumferential cracks formed a quarter of the way down from the tube’s top end. These cracks caused a fracture plane to form, resulting in the occurrence of interpenetration failure. This was accompanied by some transverse shear, which resulted in the top section of the composite tube shifting slightly to one side as interpenetration took place. This fracture plane fully formed at the initial peak load for the specimen, which was about 58.51 kN and occurred at a displacement of approximately 5.4 mm. One can see a significant drop in the load-carrying capacity as the two sections of the tube continued to interpenetrate through one another until the load dropped to a value of about 36.22 kN, at a displacement of 9.299 mm. This drop in load was accompanied by significant delamination at the fold formed due to interpenetration failure, such that part of the GFRP could be observed hanging loose from the composite tube. From this, it could be seen that the GFRP/PVC 90° composite tube specimen underwent much more delamination than the other GFRP/PVC specimens, as all the other specimens only exhibited delamination in the densification stage. Although the interpenetration and consequent drop in failure that occurred were reminiscent of Mode II failure, the GFRP/PVC 90° composite tube’s load-bearing capacity remained noticeably higher than the GFRP/PVC 45° composite tube, which underwent Mode III failure. This finding can be mainly attributed to the GFRP/PVC 90° composite tube’s fibre orientation. As the loading direction was normal to the fibre orientation, the fibres provided a higher resistance to the compression loading than the other samples, and therefore a higher load was required to overcome this resistance. However, as a direct consequence, the GFRP/PVC 90° composite tube exhibited the most severe delamination of the tested samples.

Following this, the fold formed due to interpenetration collapsed, and the two sections of the tube resulting from the split were compacted together while delamination continued to occur. The compaction of the composite tube would typically have resulted in a rise in load, while delamination weakens composite tubes and would usually cause a drop in load. However, compaction and delamination co-occurred, and, for a time, they balanced each other out, resulting in a relatively stable failure until the displacement reached about 21.22 mm. Eventually, the rise in load due to compaction became more pronounced; meanwhile, local buckling occurred at the bottom end of the tube, resulting in a rise in load up to a local maximum of approximately 50.38 kN, at a displacement of 29.05 mm. After a hinge formed due to the local buckling at the tube’s bottom end, there was a subsequent drop in load as the tube folded inwards at the hinge. After this, the two opposing failure mechanisms, compaction and delamination continued with a relatively stable load being maintained until a displacement of about 52.05 mm. Finally, in the densification stage, the amount of weakening due to delamination could not keep up with the increasing load due to compaction, and the rate at which the load-bearing capacity rose increased as the compaction progressed. After the compression tests were carried out from the specimens’ state, the GFRP/PVC 90° specimen had a noticeably higher number of loose bunches of GFRP hanging from the specimen’s other specimens. The comparatively severe fibre breakage and delamination that occurred can also be seen from the SEM image of a section of the GFRP/PVC 90° composite tube after crushing (see Figure 6d).

### 3.3. Effect of GFRP Reinforcement and Fibre Orientation

#### 3.3.1. Effect on Load-Bearing Behaviour

To observe the effect of the GFRP and fibre orientation, the load-displacement curves for all the conventional circular composite tubes with different fibre orientation angles were plotted on the same graph as the load-displacement curve for the PVC tube. The load-displacement curves shown are for the average results for these tests for each specimen configuration. Figure 10 clearly shows that all of the GFRP specimens had a considerably higher load-bearing ability than the PVC tube on its own. For a more quantifiable comparison, the PVC tube can be compared to the GFRP/PVC tube with a fibre orientation of 45°, which has the lowest minimum load in the post-crush region besides the PVC tube for the displayed curves. Based on the initial peak forces, the PVC tube’s initial peak force was 15.14 kN, while the initial peak force for the GFRP/PVC 45° composite tube was approximately 41.11 kN, more than double that of the PVC tube. In addition, if we compare the average loads for the post-crush stages for both the specimens, the PVC tube had an average load of 4.397 kN, while the GFRP/PVC 45° composite tube had an average load of about 27.16 kN, which is over six times that of the PVC tube. For the GFRP specimens, it was previously seen that local buckling and interpenetration failure were the causes of the initial failure in the tubes, and, as a result, all the tubes exhibited a noticeable drop in load-bearing capacity after the initial peak load was reached. It was observed that, as the fibre orientation angle of the tested specimens increased, the initial peak load also increased. The GFRP/PVC 90° specimen had the highest initial peak load of approximately 58.51 kN, while the GFRP/PVC 45° specimen had the lowest initial peak load of about 41.11 kN. There was an irregularity in this trend for the specimens’ mean crushing force during the post-crash stage. The mean crushing force of the GFRP/PVC 55° specimen was determined to have the lowest value during the post-crush stage of 21.68 kN. As the specimens’ orientation angle increased for the rest of the specimens, the mean crushing load also increased. The GFRP/PVC 45° composite tube had a mean crushing force of 27.16 kN for the course of the post-crushing stage, followed by the GFRP/PVC 65° specimen with a mean crushing load of 30.68 kN, and, finally, the GFRP/PVC 90° composite tube had the highest mean crushing load of 40.01 kN. Therefore, for overall load-bearing capability, the GFRP/PVC 90° composite tube performed the best. However, it was also observed that, for the GFRP/PVC 45° specimen, the displacement at which densification took place was noticeably more extensive than for the other specimens.

#### 3.3.2. Effect on Energy Absorption Capability

To determine the energy absorbed by the different composite tubes subjected to quasi-static axial compression, the area under the load–displacement curves was calculated for up to a displacement of 75 mm for all the tubes in order to have consistency in the results. Therefore, although the PVC tube experienced densification since this occurred after a displacement of 75 mm, the energy absorbed during the material densification stage was not considered. The specific energy absorbed for the composite tubes was then determined by dividing the specimens’ respective masses’ energy. As one of the main reasons for the increased use of composites as energy absorbers is their comparatively lower weights, it is essential to take mass into account when examining energy absorption capability. In addition, it is essential to remember that the sum of the energies absorbed in the pre-crush and post-crush stages is the energy that is considered useful for crashworthiness applications. The specific energy absorbed during the pre-crush, post-crush and densification stages for the tested specimen configurations is shown in the bar graph, as shown in Figure 11. Table 3 shows the vital crashworthiness parameters found during this study, including the specimens’ crush force efficiency.

Figure 11 shows the specific energy absorbed by the GFRP PVC tubes and the PVC tube on its own. The worst-performing GFRP PVC tube (i.e., GFRP/PVC 55°) had a higher value than that of the PVC tube on its own. The GFRP/PVC 55° specimen had a good specific energy absorption of 9.69 kJ kg^−1^, which was approximately 21.49% higher than the 7.98 kJ kg^−1^ value for the PVC tube on its own. For the total useful specific energy absorbed by the tested GFRP tubes, the GFRP/PVC 45° composite tube performed the best, with pre-crush and post-crush specific energy absorbed, adding up to approximately 16.47 kJ kg^−1^. Although the GFRP/PVC 45° specimen’s load-bearing capability did not stand out, due to its comparatively lower mass and delayed densification, it could perform well in energy absorption capability. The GFRP/PVC 90° specimen exhibited the next best performance with a total useful specific energy absorbed 15.05 kJ kg^−1^. Finally, the GFRP/PVC 65° specimen had a good specific energy absorption of about 13.12 kJ kg^−1^.

Table 4 compares the normalised energy absorption capability of GFRP/PVC tubes with similar tubes in the literature. The comparison was carried out based on the specific energy absorption and normalised energy absorption, considering the space volume and tubes’ cost. Among the compared tubes, GFRP/PVC tube scored the highest normalised energy absorption. However, based on the tube’s volume space’s specific energy absorption capability, the GFRP/PVC tubes are the third highest after carbon aluminium tubes.

#### 3.3.3. Effect on Crush Force Ffficiency

As shown in Table 3, the crush force efficiency for all the GFRP PVC tubes was noticeably more substantial than that of the PVC tubes. The GFRP/PVC 55°, the specimen with the lowest CFE from the reinforced specimens, had a crush force efficiency of 0.5017, which was a significant 72.82% higher than the CFE value of 0.2903 for the PVC tube. From the CFE for the remaining reinforced specimens, one can deduce that the GFRP/PVC 90° composite tube, with a CFE of 0.6838, had the most stable failure for the tested fibre orientations. However, the CFE values of 0.6607 and 0.6569 for the GFRP/PVC 45° and GFRP/PVC 65° specimens, respectively, were only slightly lower, showing that the failure of these specimens was relatively stable compared to that of the GFRP/PVC 90° composite tube. The CFE for the GFRP/PVC 45° specimen was only 3.378% less than that of the GFRP/PVC 90° specimen.

### 3.4. Fibre Orientation with the Best Performance

From the crashworthiness parameters for the tested specimens, it can be seen from the results in Table 3 that the GFRP/PVC 45° and the GFRP/PVC 90° composite tubes exhibited the best performance. Comparing these two specimens, in terms of use specific energy absorbed, the GFRP/PVC 45° specimen had a value that was approximately 8.622% higher. This result is significant as for composite tubes employed for crashworthiness applications, the specific energy absorbed is of particular importance. In terms of crush force efficiency, it was seen that the failure of these two specimens was comparable, with the GFRP/PVC 45° specimen having a CFE value that was only 3.378% lower than that of the GFRP/PVC 90° specimen. Although the GFRP/PVC 90° specimen had a significantly higher initial peak load, of 58.51 kN, as compared to that of the GFRP/PVC 45° specimen, 41.11 kN, this can be interpreted as a disadvantage when considering crashworthiness applications. As mentioned above, the initial peak load should not be high as this will help reduce the chances of passengers being injured by decreasing the size of the reaction forces experienced before the energy absorber begins to crumble. An added advantage that the GFRP/PVC 45° specimen had over its GFRP/PVC 90° counterpart was that it had a higher degree of integrity after the crushing process. It was observed that, of all the tested GFRP specimens, the GFRP/PVC 90° specimen underwent considerably more delamination, to the point that numerous fibre bunches were hanging off the specimen after compressive testing. This was attributed to the fact that this specimen’s fibre orientation was perpendicular to the compressive load’s direction. Therefore, it can be concluded that the GFRP/PVC 45° composite tube exhibited the best performance for the tested specimen configurations.

### 3.5. Cost-Effectiveness of GFRP Composite Tubes

As mentioned previously, GFRP composites are currently the most widely used type of composite due to their relative affordability. The values in Table 4 give a clearer idea of just how cost-effective GFRP composites as compared to CFRP composites [38,39]. The overall cost for the composite tubes in the table was determined using the individual material costs [34] and the following Equation(5):

(5) $$\begin{array}{c} Cost\;\left(\frac{\$}{kg}\right)=\left(Composite\;laminate\;cost\left(\frac{\$}{kg}\right)\times mass\;fraction\right)\\ +\left(Inner\;tube\;cost\left(\frac{\$}{kg}\right)\times mass\;fraction\right) \end{array}$$

A comparison was made with CFRP composites specifically, as, in automotive applications where cost is not a factor, carbon fibres are usually preferred over other reinforcement fibres due to their exceptionally high strength and stiffness [40]. The higher SEA values evidence this for the CFRP tubes in the table. However, it can be seen from the SEA/cost values in the table that, once the cost is considered, GFRP composites outperform CFRP composites by a considerable margin and are substantially more cost-effective.

## 4. Conclusions

In this study, GFRP PVC tubes with four different fibre orientations were subjected to quasi-static axial compression tests to determine fibre orientation’s effect on their crashworthiness properties. The results show that the GFRP/PVC tubes have a higher load-bearing capacity, crush force efficiency, and energy absorption capability than the PVC tube on its own. The failure mechanisms dominating the GFRP/PVC tubes’ failure mode were local buckling, transverse shear, delamination and matrix cracking. For the GFRP/PVC tubes, generally, the load-bearing, crush force efficiency and energy absorption capability improved with increasing fibre orientation angle. The GFRP/PVC 45° specimen was an exception as its crush force efficiency was second only to the GFRP/PVC 90° sample, and it had the highest specific energy absorbed value. Finally, by referring to previously conducted studies and taking material cost into account, it was shown that GFRP composites are substantially more cost-effective than CFRP composites.

Future work for this study will include an experimental and numerical investigation into the effect of trigger mechanisms on the GFRP/PVC tubes’ crashworthiness characteristics.

## Figures and Tables

**Table 1:** Material properties of composite tube constituent materials.

Constituent Property	E-Glass	Epoxy	PVC
Modulus of Elasticity (GPa)	76.00	3.200	2.414
Poisson’s Ratio	0.200	0.300	0.410
Shear Modulus (GPa)	31.00	1.100	1.000
Tensile Strength (MPa)	2410	69.00	40.69
Compressive Strength (MPa)	1750	120.0	55.17

**Table 2:** Summary of the winding values in the fabrication process.

Laminate Configuration	Winding Speed (m·min^−1^)	Feed (m·min^−1^)	Spindle Speed (RPM)
Hoop Winding (90)_8_	7.8	9.56	27
Helical Winding (65/−65)_4_	25	9.92	27
Helical Winding (55/−55)_4_	25	10.2	27
Helical Winding (45/−45)_4_	25	10.6	27

**Table 3:** Crashworthiness parameters for all tested sample configurations.

Specimen Configuration	Mass (kg)	Initial Peak Failure (kN)	Pre-Crush Energy (kJ kg^−1^)	Post-Crush Energy (kJ kg^−1)^	Densification Energy (kJ kg^−1^)	CFE
PVC	0.038 ± 0.001	15.14 ± 0.33	0.609 ± 0.044	7.37 ± 0.85	0	0.2903 ± 0.0116
GFRP/PVC@45°	0.098 ± 0.001	41.11 ± 3.11	1.181 ± 0.195	15.29 ± 1.68	5.83 ± 0.17	0.6607 ± 0.0335
GFRP/PVC@55°	0.129 ± 0.001	43.21 ± 0.29	0.460 ± 0.190	9.23 ± 2.41	12.19 ± 2.96	0.5017 ± 0.0896
GFRP/PVC@65°	0.142 ± 0.001	46.62 ± 3.34	0.615 ± 0.121	12.50 ± 1.37	14.16 ± 2.67	0.6569 ± 0.0531
GFRP/PVC@90°	0.143 ± 0.001	58.51 ± 0.64	0.852 ± 0.130	14.20 ± 1.45	14.40 ± 0.48	0.6838 ± 0.0631

**Table 4:** Energy absorption values for various circular composite tubes from literature.

Reference	Fibre	Matrix	Inner Tube Material	SEA (kJ kg^−1^)	Cost ($ kg^−1^)	SEA/Cost (kJ $^−1^)
Current	E-glass	Epoxy	PVC	16.47	2.97	5.545
[35]	E-glass	Epoxy	Aluminium	21.60	3.04	7.105
[36]	Carbon	Epoxy	Aluminium	26.87	42.74	0.629
[37]	Carbon	Epoxy	Aluminium	45.96	65.96	0.697

## Data Availability

Data is contained within the article.
